# Age and Oversizing Influence Iliac Dilatation after EVAR

**DOI:** 10.3390/jcm11237113

**Published:** 2022-11-30

**Authors:** Daphne Elisabeth Gray, Carla Samaan, Kyriakos Oikonomou, Tatjana Gruber-Rouh, Thomas Schmitz-Rixen, Wojciech Derwich

**Affiliations:** 1 Department of Vascular and Endovascular Surgery, Goethe University Hospital Frankfurt, 60590 Frankfurt, Germany; 2 Department of Radiology, Goethe University Hospital Frankfurt, 60590 Frankfurt, Germany

**Keywords:** EVAR, abdominal aortic aneurysm repair, iliac dilatation

## Abstract

In the past two decades, endovascular aortic repair (EVAR) of abdominal aortic aneurysm (AAA) has become the first line treatment for infrarenal AAA repair in many countries. While short-term results are good, concerns have been raised about long-term durability. Changes in aortoiliac anatomy, especially at the landing zones, could play a role in EVAR failure over time. The current study aimed to determine certain morphological changes in the distal iliac landing zone after EVAR implantation, as well aspossible risk factors associated with iliac sealing failure. In a retrospective analysis of a tertiary single-centre registry, including patients treated with EVAR between January 2008 and July 2018, clinical follow-up data were assessed, and computer tomography (CT) imaging was evaluated regarding morphological changes in the iliac anatomy during follow-up. For clinical analysis all patients with a minimum follow-up of one year were included; for morphological analysis of iliac anatomy all patients with available CT follow-up of a minimum of one year and a minimum of two CT scans were included. Overall, 127 out of 241 treated patients (92.1% male) were included in the clinical follow-up. Complete CT imaging of 99 iliac arteries in 55 patients was available for morphological analysis. Median postoperative follow-up (FU) for these patients was 33 months (IQR 31; min–max: 12–124). Incidence of type 1b endoleak was 3% but iliac limb detachment from the vessel wall was seen in 18.2% of the target vessels. There was a significant difference in oversizing in iliac limbs with detachment (median 13.9%, IQR 23.1) vs. without detachment (median 23.1%, IQR 19.1) (*p* = 0.034). Iliac arteries at the landing zone showed a significant diameter increase independent of an endoleak presence (overall cohort median diameter increase at one year 23.1 mm; at two years 0 mm; at three years 4.9 mm). Iliac arteries with detachment (median 14.4%; IQR 23.9) showed a significantly higher diameter increase at the landing zone after four years compared to arteries without detachment (median 5.3%; IQR 9) (*p* = 0.042). Oversizing correlated positively with an iliac diameter increase at the landing zone over time (3 m: *p*= 0.001; one year: *p* < 0.001; two years: *p* < 0.001; three years: *p* = 0.006). Older patients showed a significantly lower diameter increase at the distal landing zone over time than younger patients in the first two years after EVAR (*p* < 0.001/r = −0.606 after two years). In the current study, iliac limb oversizing was associated with increased dilatation of the distal landing zone during a three-year follow-up, while iliac limb detachment was observed less often. An older age was inversely associated to the iliac diameter increase. Future studies should clarify the association between stent graft oversizing, age, and changes in the iliac anatomy in order to identify parameters that affect EVAR durability.

## 1. Introduction

During the past two decades, endovascular aortic repair (EVAR) has gained wide acceptance as first-line treatment for abdominal aortic aneurysms (AAA). However, despite the lower early mortality in comparison to open repair, reintervention rates as well as long-term mortality appear to be higher in EVAR cases, thus raising concerns about long-term EVAR durability [1]. Changes in the aortic and iliac anatomy can be observed in patients after EVAR. Anatomic changes at the landing zone, both intrarenal and at the level of the iliac arteries may lead to failure in the sealing zone resulting in an endoleak type Ia or Ib [2,3]. Different reasons have been offered for these anatomic changes. Degeneration of the landing zone could occur due to progressive aneurysmatic disease [2], especially if the vessel at the level of the landing zone is already diseased. There are studies indicating that endovascular treatment of aortoiliac aneurysms with flared iliac limbs (limb > 16 mm diameter) could result in a higher risk of type 1b endoleak [4,5]. Falkensammer et al. analyzed the maximum diameter of 102 common iliac arteries after EVAR; there was significant dilatation of the common iliac artery (CIA) especially in already ectatic iliac arteries at the time of the operation [2]. 

The aim of our study was to analyze changes in the iliac anatomy after EVAR in relation to preoperative factors and stent-graft sizing, as well as to determine possible risk factors for EVAR failure in the distal landing zone over time.

## 2. Materials and Methods

A retrospective analysis of all patients with elective endovascular AAA repair treated from January 2008 until July 2018 was carried out. Patients with mycotic AAA and penetrating atherosclerotic ulcers as well as AAA treated with tube grafts were excluded from the analysis. For clinical data evaluation we included all patients with a minimum follow-up of 12 months in our institution. For iliac morphology analysis over time, we included all patients with at least two available CT scans with a slice thickness of ≤ 2 mm for comparison, one of which was perioperative, and one was after a minimum of one year following EVAR. All included patients initially had a distal landing zone within EVAR IFUs (diameter ≤ 25 mm; length ≥ 20 mm). Iliac arteries with a landing zone of the stent graft in the external iliac artery or iliac side branch devices were excluded from the analysis. In patients with an aortomonoiliac stent graft or a unilateral iliac side branch only the iliac side with a landing zone in the common iliac artery was included. CT angiograms were measured with GE Centricity PACS (GE Healthcare, Chalfont, UK) Software. 

The study was approved by the local ethics committee (Ethics Committee, Medical Faculty, Goethe University Hospital, Frankfurt/Main, Germany, protocol number 321/18). In agreement with the ethics committee, informed consent of the patients was waived due to the retrospective nature of the analysis.

### 2.1. Patient Selection and Follow-Up

A clinical follow-up was conducted using the hospital’s standardized AAA follow-up (FU) regime. Routinely, all EVAR patients are scheduled for a first FU visit with CT imaging three months postoperatively, followed by yearly follow-up consultations. In the early study period, yearly CT imaging was recommended for all patients; after 2015, the FU regime was changed according to the ESVS guidelines to yearly duplex ultrasound and additional CT imaging only in patients with a suspected endoleak and this took place every five years [6].

**Figure 1:**
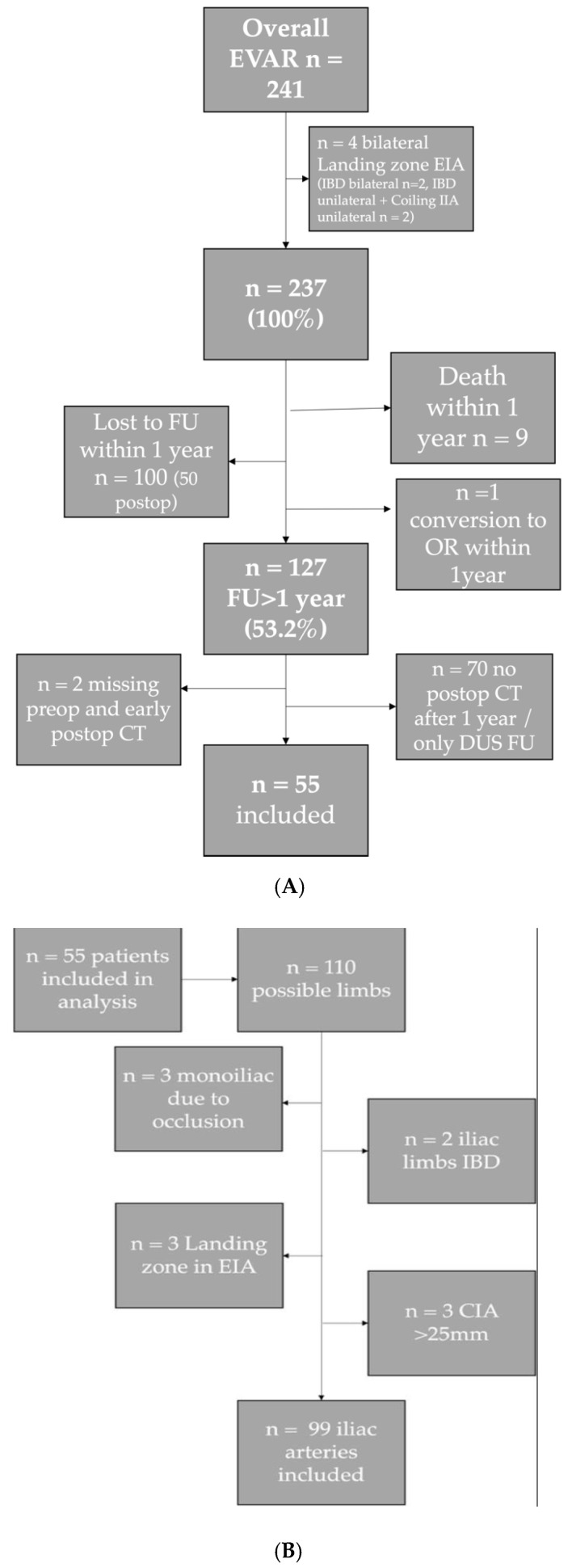
(**A**) Flow chart inclusion of patients. (**B**) Flow chart inclusion of iliac limbs. (Abbreviations: AAA = abdominal aortic aneurysm, CIA = common iliac artery, EIA = external iliac artery; IIA = internal iliac artery; IBD = iliac branch device; DUS FU = Duplex ultrasound follow-up).

From a total of 241 patients treated with aortoiliac stent grafts in the study period, 127 patients had a follow-up of over 12 months. From these 127 patients, 55 patients had available CT imaging for more than 12 months. Inclusion and exclusion reasons are shown in Figure 1A,B. Overall, 99 iliac arteries were included in the morphological analysis.

### 2.2. Measurements

**Figure 2:**
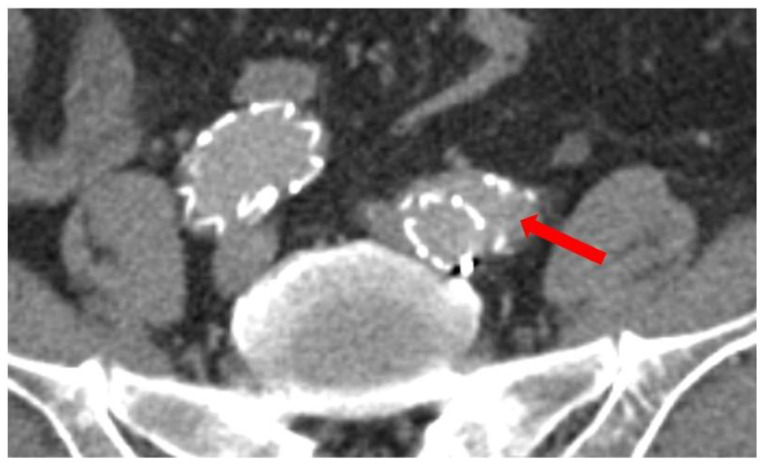
Example of an iliac detachment of the stent graft to the vessel wall at distal landing zone. Arrow pointing to the detachment with contrast medium and space between the iliac limb and the vessel wall.

All standardized measurements were performed by one of the authors (C.S.); one-third of the measurements were cross-checked by an experienced vascular surgeon (D.E.G.) with an interobserver variability of ±1 mm. The maximal diameter of the infrarenal aorta and the CIA was assessed. Additionally, we measured the diameter of the proximal CIA (10 mm distal of the aortic bifurcation), as well as the diameter at the iliac bifurcation and the diameter of the CIA at the actual landing zone of the iliac limb. With these measurements we calculated the achieved oversizing of the iliac limb. The length of the CIA was measured via a central luminal line. For iliac tortuosity evaluation we calculated the tortuosity index of the CIA (tortuosity index = centerline length/Euclidean distance) [7]. Detachment was defined as a lack of apposition of the stent graft and the native artery along the sealing stent graft in the distal landing zone (Figure 2). These measurements were performed for all follow-up CT imaging available to evaluate morphological changes in the anatomy over time.

### 2.3. Statistical Analysis

Statistics were performed using the SPSS statistics Version 27, IBM (SPSS Inc., Chicago, IL, USA) as well as BiAS. for Windows (Version 11.09, Frankfurt, Germany) and Microsoft^®^ Excel (Version 16.48, Redmond, WA, USA). Descriptive statistics are given as a median and interquartile range (IQR). Testing for normal distribution was done with the Kolmogorov–Smirnov-test as well as the Shapiro–Wilk test. For a graphic presentation we used box plot analysis. The Chi-square test following Pearson and the Fisher Exact test were used to compare the relationships between two categorical variables. The Mann–Whitney-U test was used to compare different groups in non-normal distributed data. A *p* value of <0.05 was considered as statistically significant. Correlation was calculated with the Spearman correlation for a not normally distributed metric parameter. Logistic regression analysis was performed for nominal variables. Changes in vessel diameters are shown with ladder plots. Statistics and graphs were cut off at three or five years due to a reduction in patient numbers (<10% of the collective).

## 3. Results

### 3.1. Clinical Data and Follow-Up

The median age of the cohort at the time of the operation was 72 (min–max: 55–90, IQR 12) years. 117/127 (92.1%) of the patients were male. There was no significant difference in preoperative comorbidities (arterial hypertension, COPD, PAD, diabetes, and CAD) between the 55 patients included in the morphological analysis and the rest of the cohort (*n* = 72). Median postoperative FU for all patients (*n* = 127) was 34 months (IQR 31; min–max: 12–144); for patients included in the morphological analysis median FU was 33 months (IQR 31; min–max: 12–124). Patients included in the morphological analysis (*n* = 55) had significantly higher occurrence of endoleak (40% vs. 18% (*p* = 0.006)) and higher rates of reinterventions (43.6% vs. 15.3% (*p* = 0.000)) during follow-up than patients with only a duplex ultrasound follow-up (*n* = 72). In 24 of 55 patients (43.6%) included in the analysis, eight type Ia endoleaks, three type Ib endoleaks, two type III endoleaks and 17 type II endoleaks were noted. Four patients showed more than one endoleak during FU. The mean time of endoleak diagnosis after EVAR was 6 months (min–max 3–84, IQR 18.75 months). There was one limb occlusion in the study collective which occurred concomitantly with dislocation of the limb and a type III endoleak. Endografts implanted in the 99 iliac landing zones included in the analysis were a Medtronic Endurant II^®^ (Medtronic Inc., Minneapolis, MN, USA) (*n* = 41; 74.5%), a Cook Zenith^®^ (Cook Medical Inc., Bloomington, IN, USA) (*n* = 10; 18.2%) and one patient each (1.8% each) for a Gore^®^ Excluder^®^ (W.L. Gore and Associates, Flagstaff, AZ, USA), a Vascutek Anaconda™ (Vascutek, Terumo, Inchinnan, Scotland), an Endologix Powerlink^®^ (Endologix, Inc., Irvine, CA, USA) and a Lombard Medical Aorfix™ (Lombard Medical, Inc, Irvine, CA, USA).

### 3.2. Aneurysm and Iliac Morphology

Baseline measurements for all included AAA and iliac arteries are shown in Table 1.

#### 3.2.1. AAA Aneurysm Sac

AAA aneurysm sac diameter analysis showed stable diameters over time with a mild regression in AAA diameter (median AAA sac diameter at one year −0.95%; at two years −1.42%; at 3 years −1.59% of preoperative diameter). AAA diameter showed a significant increase after one and two years (+6.26% and +15.52%) in patients with an endoleak compared to patients without endoleak (−7.41% at year 1 and −15.87% at 2 years; *p* = <0.001/<0.001).

#### 3.2.2. Maximum Diameter Common Iliac Artery

There was a significant increase in the median maximum common iliac artery diameter following the EVAR procedure (median increase 15.8 mm) within the first year which then remained stable over the follow-up (median 0 mm increase at two and three years).

#### 3.2.3. Iliac Landing Zone

**Figure 3:**
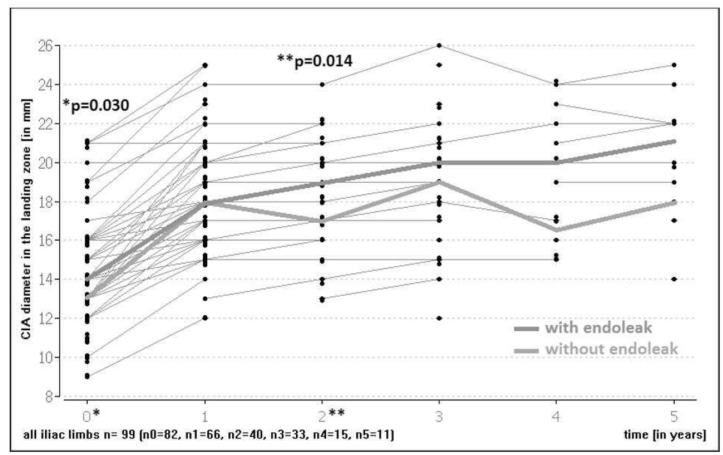
Common iliac artery (CIA) diameter in landing zone over time with endoleak vs. without endoleak. (*: baseline; **: 2 year follow-up).

There was a statistically significant iliac artery dilation at the level of the landing zone during FU (overall cohort median diameter increase at one year 23.1 mm; at two years 0 mm; at three years 4.9 mm). Patients with an endoleak had significantly wider iliac landing zones preoperatively (median 14 mm; IQR 3, min–max 9–21) than patients without an endoleak (median 13 mm; IQR 4, min–max 9–21) (*p* = 0.030). A statistically significant difference was also noted two years postoperatively in the two groups (median with an endoleak 19 mm; IQR 4; min–max 16–22 vs. without an endoleak median 17 mm; IQR 4; min–max 13.24) (*p* = 0.014) (Figure 3). However, there was no significant difference in diameter changes in the iliac landing zone in patients with an endoleak compared to patients without an endoleak over time (*p* = 0.248 at 1 year, *p* = 0.856 at two years, *p* = 1.0 at three years for percentage of growth in the landing zone compared to the first postoperative image).

### 3.3. Oversizing

Median oversizing of iliac limbs for all patients was 23.1% (min–max: 0–84.6%; IQR 18.5%). There was a statistically significant positive correlation between oversizing and dilatation of the distal landing zone at three months, one year, two and three years after EVAR (*p* = 0.001; *p* = 0.000; *p* = 0.000; *p* = 0.006/r = 0.44 for three months; 0.52 for one year; 0.59 for two years and 0.56 for a three-year FU). When using the three-month follow-up diameter as a baseline to eliminate the direct influence of the stent graft oversizing on the landing zone there was still a significant positive correlation between CIA dilatation in the landing zone and oversizing after three years (*p* = 0.021/correlation coefficient 0.61).

In our analysis, vessels with a smaller preoperative diameter at the landing zone showed higher dilatation rates at the landing zone over time. There was a significant negative correlation between the preoperative diameter at the landing zone and dilatation at the landing zone over time after three months as well as after one, two and three years (*p* = 0.001/r = −0.427; *p* = 0.000/r = −0.571; *p* = 0.002/r = −0.517; *p* = 0.009/r = −0.511). This could be explained by higher oversizing in smaller vessels. The Spearman correlation testing showed a significant negative correlation between the preoperative diameter at the landing zone and the oversizing of the iliac limb (*p* = 0.000/r = −0.493).

### 3.4. Age

Spearman correlation testing showed a statistically significant negative correlation between dilatation of the distal landing zone at a two-year FU and patient age at the time of the operation (*p* = 0.000/r = −0.606). When compared to the postoperative diameter after three months, negative correlation was seen after one year (*p* = 0.025; r = −0.326). Due to a regressing sample size over time, the analysis could only be validly evaluated for the first two years after EVAR.

### 3.5. Iliac Limb Detachment

18/99 (18.2%) iliac limbs showed detachment from the vessel wall at the distal landing zone. Detachment was seen after a median of 28 months (min–max 1–124; IQR 26). Fifteen (83%) of the detachments occurred in the first two years after EVAR. In cases of iliac limb detachment, the mean distance of the iliac limb to the vessel wall was 3 mm (min–max 1–8 mm; IQR 2). The mean age at the time of the operation for patients with detachment was 70.1 (min–max 59–84) years, the mean baseline AAA diameter was 59.9 (min–max 45–105; IQR 9.5) mm. Baseline measurements of the iliac vessels with detachment showed a median maximum CIA diameter of 15.5 (min–max 12–23; IQR 5.25) mm and at the distal landing zone 14.5 (min–max 11–21; IQR 3.5) mm. The used iliac limb had a median distal diameter of 16 (min–max 12–28; IQR 2.25). From the 18 iliac stentgrafts with detachment, nine (50%) iliac limbs were from Medtronic (Medtronic Inc., Minneapolis, MN, USA), seven (38.9) from Cook Zenith^®^ (Cook Medical Inc., Bloomington, IN, USA), one (5.6%) from Vascutek Anaconda™ (Vascutek, Terumo, Inchinnan, Scotland) and one (5.6%) limb from the Lombard Medical Aorfix™ stent graft (Lombard Medical, Inc, Irvine, CA, USA).

Only two patients (one bilateral) showed a type 1b endoleak; all three endoleaks were associated with detachment and occurred within the first six months after EVAR. Due to the small number and early occurrence of documented type Ib endoleaks, evaluation of possible risk factors for sealing failure at the distal landing zone was performed for patients with a detected detachment, a possible preliminary stage for a type 1b endoleak. For this analysis, the cohort was divided into two groups, Group I for iliac limbs with detachment and Group II without detachment.

#### Preoperative and Perioperative Factors

**Figure 4:**
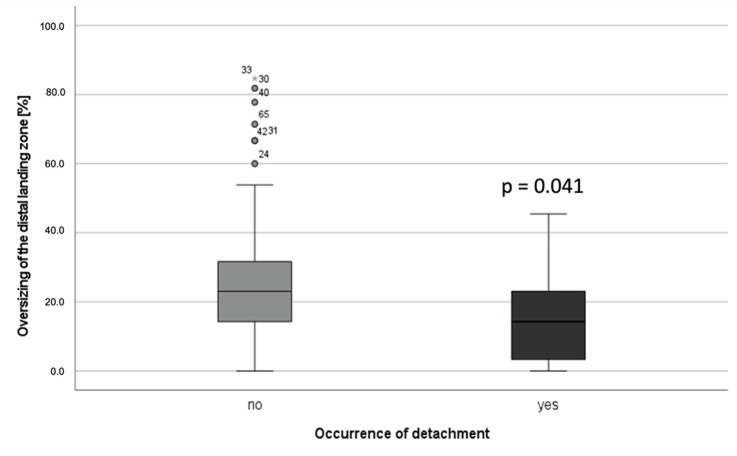
Median oversizing in patients without detachment vs. patients with detachment. (★ = extreme outliner).

Calculations of preoperative or perioperative potential risk factors are shown in Table 2. There was solely a significant difference in oversizing at the distal landing zone in patients with detachment vs. patients without detachment (*p* = 0.034). Median oversizing in patients with detachment was 13.9% (IQR 23.1) compared to 23.1% (IQR 19.1) median oversizing in patients without detachment (Figure 4).

**Figure 5:**
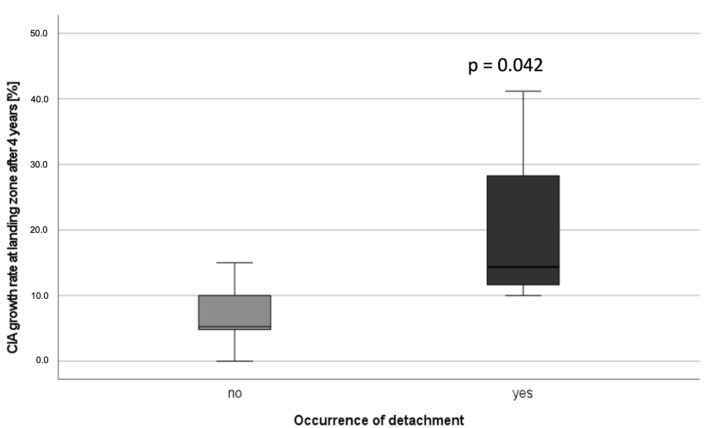
Common iliac artery growth rate at the landing zone for patients without detachment vs. patients with detachment four years after EVAR.

There was a significantly higher dilatation rate after four years compared to the diameter three months postoperatively in iliac landing zones with limb detachment (median 14.4%; IQR 23.9) compared to landing zones with no limb detachment (median 5.3%; IQR 9) (*p* = 0.042) (Figure 5).

## 4. Discussion

Our study identified several alterations in iliac anatomy over time after EVAR. While there was a slight reduction in the aneurysm sac diameter, we saw a progression in the maximal iliac artery diameter over time. This progression of the iliac diameter was seen in patients with a verified endoleak as well as in patients with no detection of an endoleak during follow-up. While the presence of any endoleak was a risk factor for higher growth of the AAA sac during the follow-up, it was not a risk factor for changes in the iliac arteries maximum diameter and at the distal landing zone. Even though the type Ib endoleak–proven failure of the distal landing zone was documented in only three cases, one in five iliac vessels showed detachment of the iliac limb from the vessel wall during follow-up, which could result in failure of the landing zone in the future. In a study by Bastos Goncalves et al. [8], the preoperative AAA diameter as well as the common iliac artery aneurysms were both considered as risk factors for distal sealing complications. Other studies have also identified aneurysmatic dilatation of the common iliac artery to be a relevant risk factor for type Ib endoleak [9,10,11]. Our study could not identify preoperative morphological risk factors for detachment over time. There was no significant difference in the patients’ age, AAA diameter, CIA diameter, landing zone length or diameter between patients with or without detachment. We could also find no statistical difference between the occurrence of detachment and no detachment in cases with CIA ≥16 mm or iliac arteries treated with iliac limbs ≥ 20 mm in the study cohort, a factor described in a previous study [4,12] as a possible risk factor for type Ib endoleak. Two out of three iliac arteries with a type 1b endoleak in our cohort had a preoperative diameter >16 mm and were treated with an iliac limb of 20 mm diameter or greater. However, we saw a significantly higher incidence of detachment in iliac arteries with lower oversizing than in iliac arteries with higher oversizing. Mascoli et al. [10] also described an association between distal oversizing <10% and the incidence of type 1b endoleak. Roos et al. [13] saw smaller oversizing in patients with reinterventions after EVAR than in patients without reinterventions (11% vs. 18%; *p* = 0.003). In contrast, there are studies indicating problems related to excessive oversizing. In a study by Sternbergh et al. [14], oversizing of more than 30% in the infrarenal landing zone showed higher incidence of stent-graft migration and aneurysm sac expansion. Other studies suggest that oversizing ≥15% in the common iliac artery is a risk factor for limb occlusion. We saw only one limb occlusion in our collective combined with complete dislocation of the limb from the main body. Other studies have already shown that there is dilatation of the iliac arteries during the follow-up after EVAR [2]. Our study confirms these changes in iliac anatomy over time, especially at the distal landing zone. While it is logical that there will be significant growth of the vessel after implantation of an oversized limb in an artery compared to the preoperative diameter, significant growth was observed when comparing the vessel diameter over time to the first postoperative measurement. There was a significant correlation between oversizing and dilatation of the common iliac artery over time. Smaller vessels showed a higher iliac growth rate after EVAR. This could be due to higher oversizing of the iliac limb in smaller vessels in our collective as shown in Figure 4. Therefore, while oversizing led to significantly less detachment in our cohort, it also resulted in more dilatation of the diameter of the iliac arteries. These study findings demonstrate the controversial role of oversizing in iliac limbs. While higher oversizing showed less detachment of the distal iliac limb in the mid-term follow-up, it nevertheless induced iliac dilatation over time. Longer CT follow-up data could possibly identify which “risk” outweighs the other and help find the “perfect fit” for the oversizing of iliac limbs. Another interesting factor that seems to influence vessel dilatation over time is age. In the first two years after EVAR, younger patients showed significantly more dilatation of the vessel within the first two years compared to older patients. Similar results were shown in a study by Telles et al. [15]. Our study suggests that next to oversizing, age also plays a possible role in changes in the iliac anatomy. If younger patients show a higher iliac vessel growth rate, these patients could be more at risk of secondary complications due to dilatation of the landing zone. This weighs into the general discussion whether young, fit AAA patients should be treated primarily with EVAR or open repair. Unfortunately, comparison of the natural history of iliac vessels after open repair and EVAR is difficult due to the lack of reliable imaging data.

The main limitation of our study is the small study collective and possible selection bias. There were two main reasons. Compliance in EVAR surveillance is a known problem. Looking at the literature, after five years, fewer than 50% of the patients treated with EVAR attended routine follow-up appointments [16]. While 44% of our patients were fully compliant with our EVAR follow-up schedule after the operation, 21% did not attend any further follow-ups after the operation and 25% were lost to the follow-up while still alive. Thirty percent of the patients died over time. The second main reason is the limited number of CT scans after EVAR. Due to missing CT data, we could include only one fourth of our overall EVAR collective. Objective evaluation and measurement of the iliac anatomy are only feasible with adequate CT scans. Since 2015, in accordance with ESVS guidelines and in order to reduce radiation and contrast the medium exposure of our patients, only patients with suspicious findings in the duplex ultrasound or contrast enhanced ultrasound underwent additional postoperative CT angiograms. This may have led to an overreporting of the incidence of endoleaks in our collective. Comparing our patients included in the morphological analysis (*n* = 55) with the ones with an available clinical follow and ultrasound follow-up, there were significantly higher rates of an endoleak as well as reinterventions in patients that were included in the measurements. Nevertheless, while an endoleak was a risk factor for AAA sac growth, it did not affect iliac growth over time. Additionally, the positive correlation between oversizing and growth in the iliac artery diameter at the landing zone as well as the negative correlation between age and iliac artery growth after two years were also seen in the subgroups analysis of iliac arteries in patients with no endoleak. Therefore, this selection bias with a high number of endoleak patients may not be too relevant when evaluating changes in iliac anatomy. In conclusion, our sample size did not allow for comparisons of the effect of different endografts on iliac artery dilatation. Most of our patients were treated with polyester stent grafts (92.7%), but we could not evaluate whether different graft materials and the radial force of the stent graft influence the dilatation at the landing zone.

## 5. Conclusions

Our study confirmed that significant changes in the iliac anatomy occur after EVAR. There is a significant correlation between oversizing and dilatation of the common iliac artery over time at the landing zone. Younger patients showed more rapid changes in the iliac vessel diameter compared to older patients in the first two years after EVAR. Almost 20% of our collective showed detachment of the distal limb over time, which could lead to future type 1b endoleak. Patients with a greater oversizing of the iliac limb showed lower limb detachment rates. Oversizing appears to be a key element in changes in the iliac anatomy: the right balance between sufficient oversizing to prevent detachment and not excessive oversizing that induces iliac dilatation must be found. Our findings also suggest a long-term follow-up is important to detect possible complications due to vessel growth at the landing zone after EVAR.

## Figures and Tables

**Table 1:** Baseline measurements.

Measurements	Median (mm)	IQR (Min–Max)
Preoperative measurements		
max. AAA diameter	56.5	11.25
diameter aortic bifurcation	27	8.5 (20–55)
proximal CIA diameter	14	3.25 (10–22)
max. CIA diameter	16	4 (11–30)
distal CIA diameter	15	4 (11–22)
CIA diameter at landing zone	14	3 (9–21)
diameter proximal EIA	10	2 (6–15)
length centerline CIA	62	18.75 (26–97)
length Euclidian CIA	53	18 (26–88)
calculated tortuosity index	1.1	0.15 (1.0–1.79)
Early postoperative measurement		
distance distal end of stent graft to iliac bifurcation	15	19.5 (1–42)

(Abbreviations: AAA = abdominal aortic aneurysm, CIA = common iliac artery, EIA = external iliac artery).

**Table 2:** Potential risk factors for detachment.

Potential Preoperative/Perioperative Risk Factors	*n*	Group I Median (IQR; Min.–Max.)	Group II Median (IQR; Min.–Max.)	*p*
Preoperative max AAA diameter [in mm]	82	56.5 (9.5; 45–105)	56 (11; 45–88)	0.781
Preoperative max CIA diameter [in mm]	82	15.5 (5.25; 12–23)	16 (4; 11–30)	0.857
Preoperative max. diameter iliac bifurcation [in mm]	82	15 (5; 11–22)	15 (3.5; 10–23)	0.646
Preoperative diameter distal landing zone [in mm]	82	14.5 (3.5; 11–21)	14 (3; 9–21)	0.345
Diameter of iliac limb [in mm]	97	16 (2.25; 12–28)	16 (4; 13–28)	0.507
Oversizing at distal landing zone [in %]	80	13.91 (23.1; 0–45.5)	23.08 (19.1; 0–84.6)	0.034
Preoperative length of CIA [in mm]	77	53 (18.8; 37–94)	63 (18; 26–97)	0.055
Tortuosity index of CIA	77	1.13 (0.2; 1–1.4)	1.1 (0.2; 1–1.8)	0.248
Distance end of iliac limb to iliac bifurcation [in mm]	68	20 (20; 2–38)	19 (21; 1–51)	0.938
Patients age at time of EVAR [in years]	99	69 (8.3; 59–84)	70 (13; 57–90)	0.996

(Abbreviations: AAA = abdominal aortic aneurysm, CIA = common iliac artery).

## Data Availability

The data presented in this study are available on request from the corresponding author. The data are not publicly available due to ethical and privacy compliance.
